# A Case of Zieve’s Syndrome With the Development of Delirium Tremens

**DOI:** 10.7759/cureus.37225

**Published:** 2023-04-06

**Authors:** Luis M Nieto, John P Martinez, Sharon Narvaez, Cinna Attar, Thomas Lall

**Affiliations:** 1 Internal Medicine, Wellstar Cobb Medical Center, Austell, USA; 2 Escuela de Medicina, Universidad de Guayaquil, Guayaquil, ECU; 3 Hospital Medicine, Wellstar Spalding Medical Center, Griffin, USA

**Keywords:** delirium tremens, acute liver injury, alcohol withdrawal, hyperlipidemia, hemolysis

## Abstract

A 50-year-old man presented to the emergency department with dark urine and altered mental status. Upon examination, the patient was found to be jaundiced with normal vitals. Laboratory investigation demonstrated macrocytic anemia and abnormal liver function tests. During his hospitalization, he developed delirium tremens in addition to the discovery of acute hemolytic anemia, hypercholesterolemia, and hypertriglyceridemia. Therefore, he was diagnosed with Zieve’s syndrome (ZS), a rarely reported disease characterized by hemolytic anemia, cholestatic jaundice, and transient hyperlipidemia. Physicians encountering acute hemolytic anemia in a patient with concomitant acute liver injury should consider ZS as a differential diagnosis, as prompt recognition of the syndrome can help prevent unnecessary procedures and therapy.

## Introduction

Zieve’s syndrome (ZS) is a rare condition that occurs in the setting of alcohol-related liver disease, characterized by hemolytic anemia, hyperlipidemia, and jaundice, especially found in heavy alcohol users after a binge drink [1,2]. Hyperlipidemia can be challenging as levels can fluctuate and, in certain cases, lipid levels can be normal, which is an atypical presentation of this syndrome [2]. The clinical manifestation of ZS is nonspecific and patients may present with nausea, abdominal pain, jaundice, and other vague symptoms [2]. This syndrome is difficult to differentiate from other alcohol-related conditions, resulting in unnecessary therapies, diagnostic interventions, and prolonged hospital stays, directly impacting healthcare costs and morbidity [3].

## Case presentation

A 50-year-old man with a 30-year history of alcohol use, heavy drinking in the past six years (16 ounces of vodka daily), previous hospitalizations for alcohol intoxication, and withdrawal presented to the ED with altered mental status (somnolence and confusion) for the past 12 hours and dark urine of two-week duration. Initial vital signs were normal and physical examination only revealed jaundice with normal liver and spleen size and a BMI of 22.6 kg/m^2^. Laboratory results were notable for macrocytic anemia and abnormal liver function tests with elevated aspartate aminotransferase (AST), alanine aminotransferase (ALT), alkaline phosphatase (ALP), and total bilirubin (TB). Important results to assess the liver's biosynthetic capacity were abnormal, with decreased albumin and elevated prothrombin time (PT)/international normalized ratio (INR). His ethanol level was markedly elevated and consistent with his routine binge drinking. In addition, he had an abnormal lipid panel with elevated total cholesterol (TC), triglyceride (TG), and low-density lipoprotein (LDL). Labs are outlined with details in Table 1. Hepatitis viral panel showed negative results. Labs from an admission six months ago due to alcohol intoxication showed AST at 224 IU/L, ALT at 84 IU/L, ALP at 74 IU/L, TB at 1.0 mg/dL, hemoglobin (Hgb) at 13.0 g/dL, platelets at 131 10E9/L, and normal renal function/electrolytes. No previous lipid panel was found in his records.

**Table 1:** Laboratory results at admission

	Results	Normal values
Hemoglobin	12.0 g/dL	14.0-18.0 g/dL
Hematocrit	37.5%	42.0-52.0%
Platelets	153 10E9/L	150-450 10E9/L
Mean corpuscular volume	103.9 fL	80.0-94.0 fL
Aspartate aminotransferase	291 IU/L	13-39 IU/L
Alanine aminotransferase	91 IU/L	7-52 IU/L
Alkaline phosphatase	212 IU/L	34-104 IU/L
Total bilirubin	14.6 mg/dL	0.0-1.0 mg/dL
Direct/indirect	8.5 mg/dL/6.1 mg/dL	0.0-0.2 mg/dL
Albumin	3.2 d/dL	3.5-5.7 g/dL
Prothrombin time	16.2 sec	10.3-13.3 sec
International normalized ratio (INR)	1.37	0.85-1.15 ratio
Ethanol	322 mg/dL	0-20 mg/dL
Blood urea nitrogen (BUN)	10 mg/dL	7-25 mg/dL
Creatinine	0.85 mg/dL	0.70-1.30 mg/dL
Anion gap	17	2-11
Sodium	133 mmol/L	136-145 mmol/L
Potassium	4.0 mmol/L	3.5-5.1 mmol/L
Magnesium	1.8 mg/dL	1.9-2.7 mg/dL
Calcium	8.0 mg/dL	8.6-10.3 mg/dL
Lactic acid	3.8 mmol/L	0.5-2.0 mmol/L
Total cholesterol	416 mg/dL	<200 mg/dL
Triglyceride	394 mg/dL	0-149 mg/dL
Low-density lipoprotein	329 mg/dL	75-193 mg/dL
Ammonia	26 umol/L	16-53 umol/L

**Figure 1:**
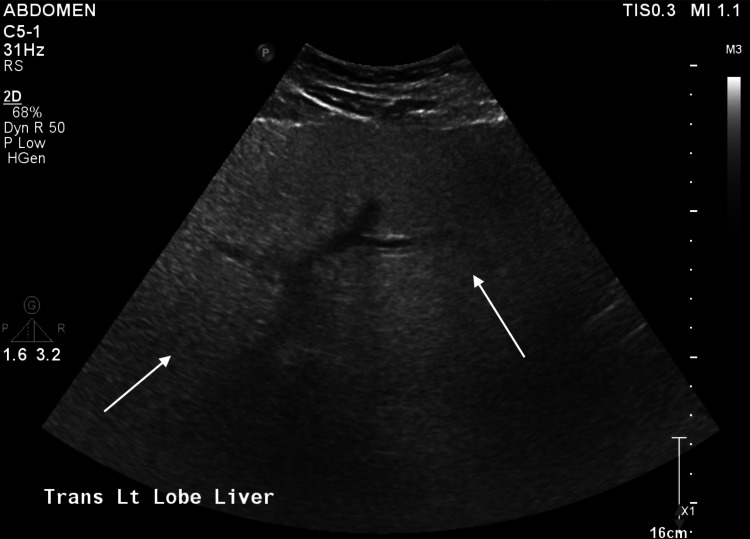
Liver ultrasound with hepatic steatosis

Abdominal ultrasound (US) was found to be consistent with hepatic steatosis, noticing the absence of the normal echogenic walls of the portal veins and hepatic veins (Figure 1). A magnetic resonance cholangiopancreatography (MRCP) showed no choledocholithiasis or extra/intrahepatic biliary dilatation.

The model for end-stage liver disease-sodium (MELD-Na) score was 26 and Maddrey's discriminant function (MDF) for alcoholic hepatitis was 26. He developed delirium tremens after 72 hours, managed with lorazepam 4 mg pro re nata (PRN), chlordiazepoxide 25 mg every six hours, and supportive care, which was mainly abstinence and vitamin C/B solution. Without signs of active bleeding, his Hgb dropped to 9.9 g/dL and TB peaked at 21.3 mg/dL with a calculated MDF greater than 32. Steroids were initiated for acute alcoholic hepatitis but discontinued after increased suspicion for ZS and calculation of a Lille model score of 0.607 points at seven days with TB at 19.2 mg/dL. Hemolysis was confirmed with low haptoglobin and elevated reticulocytes. The complete workup is summarized in Table 2. The patient was discharged after a two-week hospital stay with improved labs outlined in Table 3. Unfortunately, the patient did not present for follow-up as planned after hospital discharge.

**Table 2:** Hemolysis workup

	Results	Normal values
Lactate dehydrogenase	159 IU/L	140-271 IU/L
Haptoglobin	<30 mg/dL	44-215 mg/dL
Reticulocytes	5.5%	0.50-1.50%
Fibrinogen	359 mg/dL	140-420 mg/dL
Fibrin monomer	Negative	Negative
Direct antiglobulin	Negative	Negative
Direct Coombs test	Negative	Negative

**Table 3:** Lab results at discharge

	Results	Normal values
Aspartate aminotransferase	77 IU/L	13-39 IU/L
Alanine aminotransferase	72 IU/L	7-52 IU/L
Alkaline phosphatase	157 IU/L	34-104 IU/L
Total bilirubin	13.5 mg/dL	0.0-1.0 mg/dL
Total cholesterol	354 mg/dL	<200 mg/dL
Triglyceride	244 mg/dL	0-149 mg/dL
Low-density lipoprotein	295 mg/dL	75-193 mg/dL

## Discussion

ZS is a rare disease among chronic alcohol abusers. It is characterized by the triad of Coombs-negative hemolytic anemia, cholestatic jaundice, and transient hyperlipidemia in patients with a history of alcohol abuse and liver disease [1]. The mechanism of hemolysis is unclear, but it has been related to an accumulation of polyunsaturated fatty acid and cholesterol inside RBC membranes [3,4]. These changes cause severe hyperbilirubinemia in ZS due to several factors, including hepatocellular damage and hemolysis [4]. Furthermore, other studies have suggested that a deficiency in vitamin E induced by alcohol can aggravate the hemolytic process [5]. Therefore, the associated severe hemolysis seen in ZS can be inappropriately used to overestimate morbidity and mortality when calculating MDF, based on the abnormally high TB level, without considering other etiologies [6,7]. However, it has also been reported that patients with a deficiency of lipoprotein lipase can be predisposed to transient hyperlipidemia in more than 50% of patients with ZS [3]. MDF is an excellent evidence-based tool to assist in risk stratification among patients with alcoholic hepatitis; it improves outcomes based on the disease severity and helps establish the use of specific interventions [8]. This calculation uses PT and TB to predict short-term mortality. MDF scores < 32 indicate mild to moderate alcoholic hepatitis and do not indicate a benefit from the introduction of steroids. ZS treatment consists of alcohol abstinence, hydration, and nutrition [8]. Pharmacologic therapy with a glucocorticoid is not recommended in patients with mild to moderate alcoholic hepatitis. MDF score > 32 indicates severe alcoholic hepatitis, and will likely benefit from treatment including glucocorticoid or pentoxifylline [8].

Our case presents a patient with characteristics similar to acute alcoholic hepatitis (AAH) with non-specific symptoms of jaundice, elevated bilirubin, elevated hepatic enzymes, abnormal INR, and abnormal lipid panel, in addition to an ultrasound consistent with hepatic steatosis [1]. However, it is essential to consider that ZS can also present with non-specific symptoms besides the classic triad. Symptoms like nausea, vomiting, abdominal pain, weakness, and low-grade fever can mimic AAH [3,9]. However, a significant difference is that AAH presents with macrocytic anemia, whereas ZS causes non-autoimmune acute hemolytic anemia [5,6].

During our patient's first encounter, he presented with a history of alcohol abuse and intoxication. Labs were consistent with liver injury and macrocytic anemia characteristic of AAH. After three days, the patient decompensated, developing severe withdrawal with features consistent with delirium tremens (DT). After the development of DT, laboratory studies demonstrated worsening of liver function tests, and MDF calculation increased to 32, leading us to initiate treatment for AAH with corticosteroids. Studies have shown that patients with AAH and an MDF score of >32 have high short-term mortality, estimated to be about 25% to 45% at one month [8]. Data have demonstrated that using corticosteroids has a short-term outcome relative risk reduction in mortality by 15% to 63% [10]. Short-term outcomes can vary depending on the severity of the patient's condition. Overtreatment of ZS using steroids can lead to unnecessary complications like infections and gastrointestinal complications; corticosteroids are more useful for autoimmune hemolytic anemia, but this may not be true for ZS [5]. After witnessing a lack of improvement in our patient with an elevated Lille model score, we discontinued the corticosteroid, providing only supportive care. Lille model scores help to provide early recognition of patients with severe AAH who are not responding to corticosteroids. Lille scores > 0.45 suggest that a patient is not responding to steroids. On the other hand, a score of <0.45 correlates to corticosteroid response [8].

In addition to the unnecessary treatment and complementary tests due to misdiagnosis, a patient can also experience prolonged hospitalization increasing the burden of healthcare costs and possible harm to the patient [11,12].

## Conclusions

ZS is an underreported syndrome, usually diagnosed late after undergoing unnecessary treatment and supplementary testing. This case report aims to raise awareness among physicians to recognize ZS in alcoholic patients with hemolytic anemia and avoid unnecessary diagnostic workups and therapy. Also, as this syndrome develops in the context of alcohol abuse, patients may develop alcohol withdrawal during hospitalization. ZS and its association with DT should be suspected in patients with a triad of hemolytic anemia, unexplained jaundice, and hyperlipidemia in the setting of acute alcohol intake. Future studies should be considered to evaluate the association between ZS and DT.
